# Ursolic Acid-Enriched Herba Cynomorii Extract Induces Mitochondrial Uncoupling and Glutathione Redox Cycling Through Mitochondrial Reactive Oxygen Species Generation: Protection Against Menadione Cytotoxicity in H9c2 Cells

**DOI:** 10.3390/molecules19021576

**Published:** 2014-01-27

**Authors:** Jihang Chen, Hoi Shan Wong, Kam Ming Ko

**Affiliations:** Division of Life Science, Hong Kong University of Science and Technology, Clear Water Bay, Hong Kong SAR, Hong Kong, China

**Keywords:** *Cynomorium songaricum*, ursolic acid, mitochondrial uncoupling, glutathione redox cycling, oxidant injury

## Abstract

Herba Cynomorii (*Cynomorium songaricum* Rupr., Cynomoriaceae) is one of the most commonly used ‘Yang-invigorating’ tonic herbs in Traditional Chinese Medicine (TCM). An earlier study in our laboratory has demonstrated that HCY2, an ursolic acid-enriched fraction derived from Herba Cynomorii, increased mitochondrial ATP generation capacity (ATP-GC) and induced mitochondrial uncoupling as well as a cellular glutathione response, thereby protecting against oxidant injury in H9c2 cells. In this study, we demonstrated that pre-incubation of H9c2 cells with HCY2 increased mitochondrial reactive oxygen species (ROS) generation in these cells, which is likely an event secondary to the stimulation of the mitochondrial electron transport chain. The suppression of mitochondrial ROS by the antioxidant dimethylthiourea abrogated the HCY2-induced enhancement of mitochondrial uncoupling and glutathione reductase (GR)-mediated glutathione redox cycling, and also protected against menadione-induced cytotoxicity. Studies using specific inhibitors of uncoupling protein and GR suggested that the HCY2-induced mitochondrial uncoupling and glutathione redox cycling play a determining role in the cytoprotection against menadione-induced oxidant injury in H9c2 cells. Experimental evidence obtained thus far supports the causal role of HCY2-induced mitochondrial ROS production in eliciting mitochondrial uncoupling and glutathione antioxidant responses, which offer cytoprotection against oxidant injury in H9c2 cells.

## 1. Introduction

The mitochondrion serves as a platform for cellular energy metabolism as well as signal transduction pathways relevant to the regulation of cell survival and death [1]. Mitochondrial dysfunction is believed to be pathologically related to various forms of cardiovascular diseases such as heart failure [2]. The maintenance of mitochondrial functional integrity and antioxidant capacity is therefore critical for cell survival, particularly for cardiac cells under oxidative stress conditions. In this connection, ‘Yang-invigoration’ in Traditional Chinese Medicine (TCM), which is pharmacologically viewed as the enhancement of mitochondrial ATP generation and the associated increase in mitochondrial antioxidant capacity, may offer a promising prospect for preventing oxidant injury in the heart [3].

Herba Cynomorii (the fresh stem of *Cynomorium songaricum* Rupr., Cynomoriaceae; also known as Suo-Yang in Chinese) is one of the most commonly used ‘Yang-invigorating’ Chinese tonic herbs. This herb is mainly found distributed across the deserts in Western China, such as Xinjiang, Qinghai, Gansu, Ningxia, Inner Mongolia and Shanxi, as well as in Central Asia, Iran and Mongolia. In TCM practice Herba Cynomorii is prescribed for treating lumbar weakness and enhancing sexual ability for both men and women. In recent years, the major constituents of Herba Cynomorii have so far been identified as phenolic compounds, steroids, triterpenes, among other constituents [4]. In addition, pharmacological investigations have shown that certain fractions and chemical compounds isolated from Herba Cynomorii possess a wide spectrum of biological activities, including anti-HIV protease [5], anti-HCV protease [6], anti-apoptosis, antioxidant, anti-aging, anti-fatigue, anti-osteoporotic, anti-diabetic and fertility promoting actions [7,8]. A previous study in our laboratory has demonstrated that HCY2, an ursolic acid (UA) -enriched fraction derived from Herba Cynomorii increased mitochondrial ATP generation capacity (ATP-GC) and induced mitochondrial uncoupling as well as a cellular glutathione response, resulting in protection against oxidant injury in H9c2 cells [9]. However, the biochemical mechanism underlying the induction of these cellular protective responses by HCY2 is still unknown. With reference to other experimental findings on Yang-tonic herbs or formulas [10,11], we hypothesized that ‘Yang-invigoration’ induced by HCY2 may involve a sustained and low level of mitochondrial reactive oxygen species (ROS) production, which is secondary to the increased activity of the electron transport chain [12]. The increased formation of ROS within mitochondria would further trigger cellular responses, including mitochondrial uncoupling and glutathione redox cycling [13], with resultant protection against oxidant injury. To test this hypothesis, we examined the effect of HCY2 on mitochondrial ROS production and provided evidence for the supporting the role of mitochondrial ROS in HCY2-induced mitochondrial uncoupling and glutathione redox cycling in H9c2 cells. The effects of an antioxidant and specific inhibitors of uncoupling protein (UCP) and glutathione reductase (GR), an enzyme for catalyzing the regeneration of reduced glutathione (GSH) from oxidized glutathione (GSSG), on HCY2-induced protection against menadione-induced cytotoxicity were also investigated.

## 2. Results and Discussion

**Figure 1:**
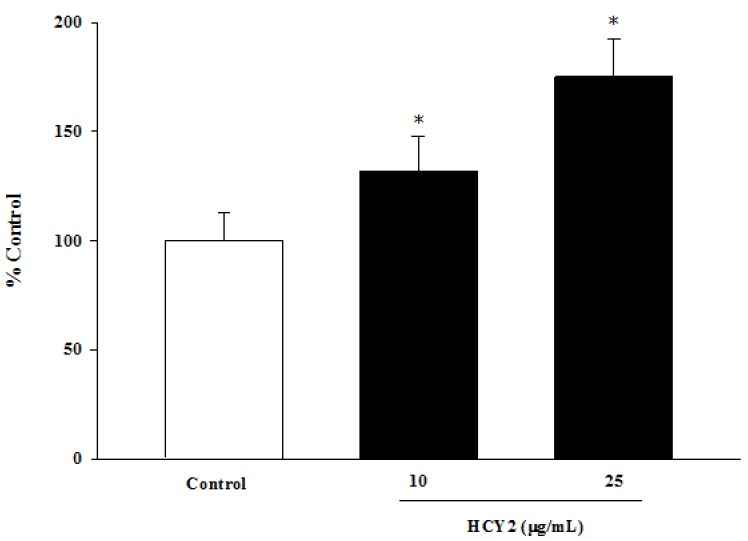
Effects of pre-incubation of H9c2 cells with HCY2 on mitochondrial electron transport. Cells were pre-incubated with HCY2 (10 or 25 µg/mL) for 4 h. Pyruvate and malate-supported mitochondrial electron transport was measured by MTT reduction, as described in the Experimental Section. Data are expressed as the percentage of non-herbal extract pre-incubated control values (0.072 ± 0.009). Each bar represents the mean ± SD, with *n* = 6. * Significantly different from the control group without HCY2 pre-incubation (*p* < 0.05).

The extent of eletron transport in isolated mitochondria was measured by monitoring the reduction of MTT. As shown in Figure 1, pre-incubation of H9c2 cells with HCY2 concentration-dependently increased the extent of mitochondrial electron transport supported by pyruvate and malate, with the extent of stimulation being 32% and 75% relative to control at 10 and 25 μg/mL, respectively. 

**Figure 2:**
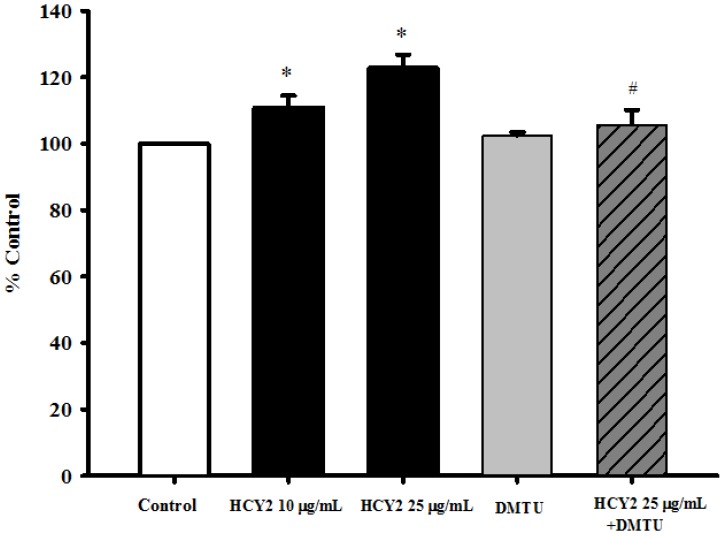
Effects of HCY2 on mitochondrial ROS production in H9c2 cells. Cells were pre-incubated with HCY2 (10 or 25 µg/mL) with or without DMTU (20 mM) for 4 h. Mitochondrial ROS production was measured as described in the Experimental Section. Values given are mean ± SD, with *n* = 3. * Significantly different from the control group without HCY2 pre-incubation; # significantly different from the HCY2-pre-incubated group (*p* < 0.05).

As shown in Figure 2, the HCY2 (10 or 25 µg/mL)-induced stimulation of mitochondrial electron transport was accompanied by a small but significant increase in mitochondrial ROS production (11% and 23% respectively). Although DMTU (a thiol-containing antioxidant; 20 mM) *per se* did not alter mitochondrial ROS production, co-incubation of HCY2 (25 µg/mL) with DMTU almost completely abrogated the HCY2-induced mitochondrial ROS production in H9c2 cells.

**Figure 3:**
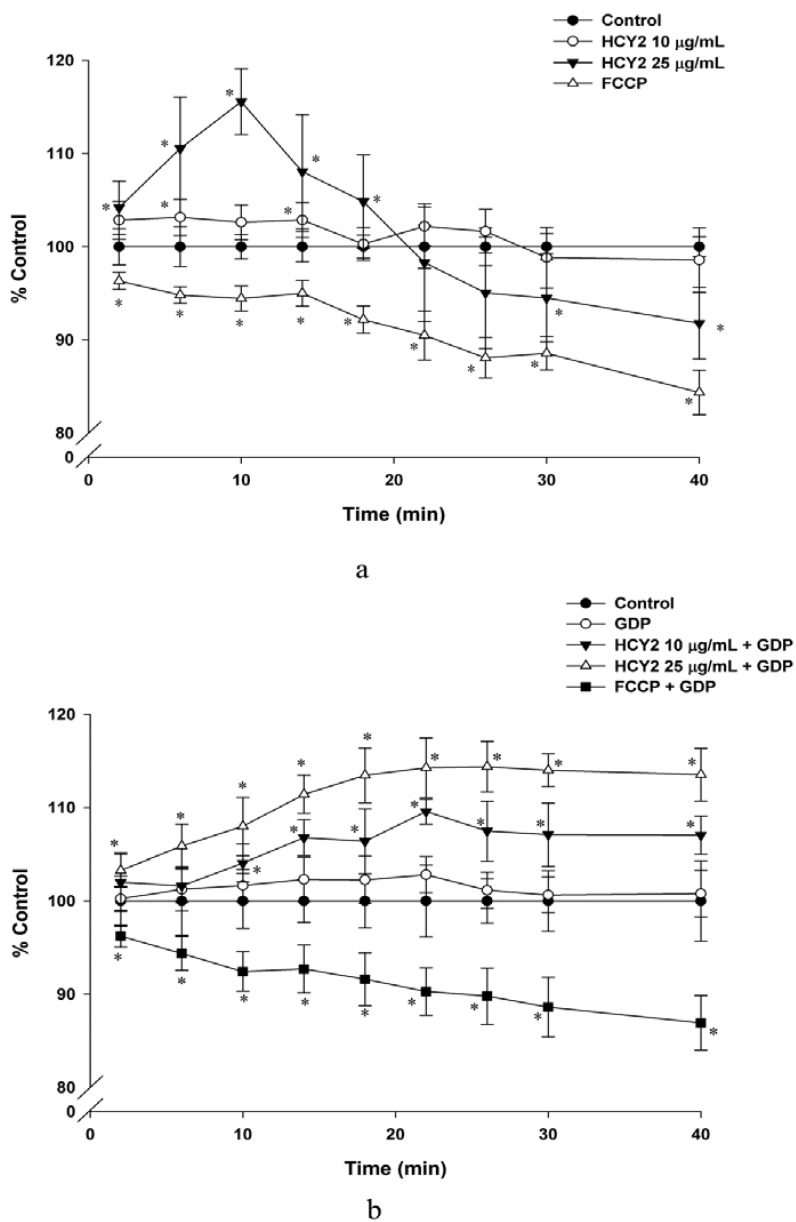
Effects of HCY2 on mitochondrial membrane potential in H9c2 cells. Cells were incubated with HCY2 (10 or 25 µg/mL) without (**a**) or with (**b**) GDP (500 µM). FCCP (100 µM), a chemical uncoupler, was used as a positive control in this experiment. Mitochondrial membrane potential was measured as described in the Experimental Section. Data are expressed as percent control with respective to the time-matched non-herbal extract pre-incubated control. Values given are mean ± SD, with *n* = 3. * Significantly different from the control group without HCY2 pre-incubation (*p* < 0.05).

To investigate whether the stimulation of mitochondrial electron transport is accompanied by increases in mitochondrial membrane potential, the changes in mitochondrial membrane potential were monitored. Figure 3a shows that incubation of H9c2 cells with HCY2 (25 µg/mL) caused time-dependent increases in mitochondrial membrane potential during the initial time period (0–10 min), with the maximum extent of stimulation being 16% at 10 min. Interestingly, there were gradual decreases in mitochondrial membrane potential following the 10-min incubation with HCY2 (25 µg/mL), with the extent of decrease being 8% at 40 min, when compared with the vehicle control. However, FCCP, a chemical uncoupler of mitochondria, was found to cause a gradual decrease in mitochondrial membrane potential, the extent of reduction being approximately 16% at the end of the 40-min incubation. As shown in Figure 3b, guanosine diphosphate (GDP, a UCP inhibitor) did not affect the mitochondrial membrane potential when compared with the control group. The co-incubation of the H9c2 cells with GDP during the exposure to HCY2 was found to inhibit the decreases in mitochondrial membrane potential at both tested concentrations of HCY2. It should be noted that the co-incubation with GDP did not affect the FCCP-induced decreases in mitochondrial membrane potential.

Table 1 shows that pre-incubation of H9c2 cells with HCY2 at 25 µg/mL not only increased state 3 respiration (by 41%), but also stimulated state 4 respiration (by 78%). Consequently, the ratio of state 3 to state 4 respiration rate, which is also referred to as the ‘respiration control ratio’ (RCR, an index of mitochondrial coupling efficiency), was decreased (by 20%). The uncoupling effect induced by HCY2 (25 µg /mL) was completely abrogated by GDP. To explore the role of ROS in HCY2-induced mitochondrial uncoupling, H9c2 cells were co-incubated with HCY2 (25 µg/mL) and DMTU (20 mM), and mitochondrial respiration was measured. The result showed that DMTU *per se* did not alter mitochondrial respiration when compared with the control group. Although co-incubation with DMTU did not affect the HCY2-induced increase in state 3 respiration, the HCY2-induced stimulation of state 4 respiration was almost completely suppressed by DMTU. Consequently, DMTU co-incubation completely abrogated the HCY2-induced mitochondrial uncoupling, as indicated by the increased RCR value.

**Figure 4:**
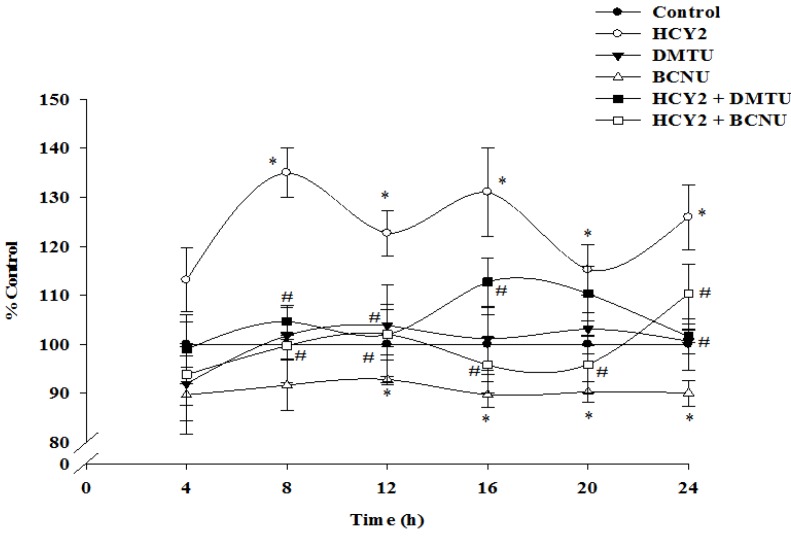
Effects of DMTU and BCNU on HCY2-induced enhancement on the glutathione redox cycling in H9c2 cells. Cells were incubated with HCY2 (25 µg/mL) with or without DMTU (20 mM) or BCNU (50 µM). Cellular GSH levels were measured as described in the Experimental Section. Data are expressed as the percentage of non-herbal extract or non-drug pre-incubated parallel control values (initial control GSH level = 25.01 ± 1.96 nmol/mg protein) at various periods of herbal extract or drug incubation. Values given are means ± SD, with *n* = 3. * Significantly different from the control group without HCY2 pre-incubation; # significantly different from the HCY2-pre-incubated group (*p* < 0.05).

As shown in Figure 4, incubation of H9c2 cells with HCY2 at 25 µg /mL produced time-driven cyclic variations in cellular GSH levels, with the increase in the amplitude of oscillation being 35%, when compared with the control. While DMTU (20 mM) did not produce any detectable changes in cellular GSH levels, BCNU (50 μM) significantly decreased the cellular GSH levels when compared with the control group. The HCY2-induced time-driven cyclic variation of cellular GSH level, referred to as glutathione redox cycling, was largely abrogated by DMTU and BCNU.

**Table 1:** Effects of HCY2 on mitochondrial respiration in H9c2 cells in the absence or presence of GDP or DMTU. * Significantly different from the control group without HCY2 pre-incubation; # significantly different from the HCY2-pre-incubated group (*p* < 0.05)

Respiration rate	Control	HCY2	GDP	HCY2 + GDP	DMTU	HCY2 + DMTU
State 3	100.00 ± 3.02	145.70 ^*^ ± 12.48	-	-	104.27 ± 5.68	139.22 ^*^ ± 3.86
State 4	100.00 ± 3.77	177.80 ^*^ ± 13.22	98.68 ± 2.47	95.45 ^#^ ± 3.27	102.04 ± 5.93	107.64 ^#^ ± 4.88
State 3/State 4	100.00 ± 1.88	81.83 ^*^ ± 2.57	101.23 ± 4.31	152.86 ^*#^ ± 13.64	102.10 ± 0.54	129.73 ^*#^ ± 6.80

**Figure 5:**
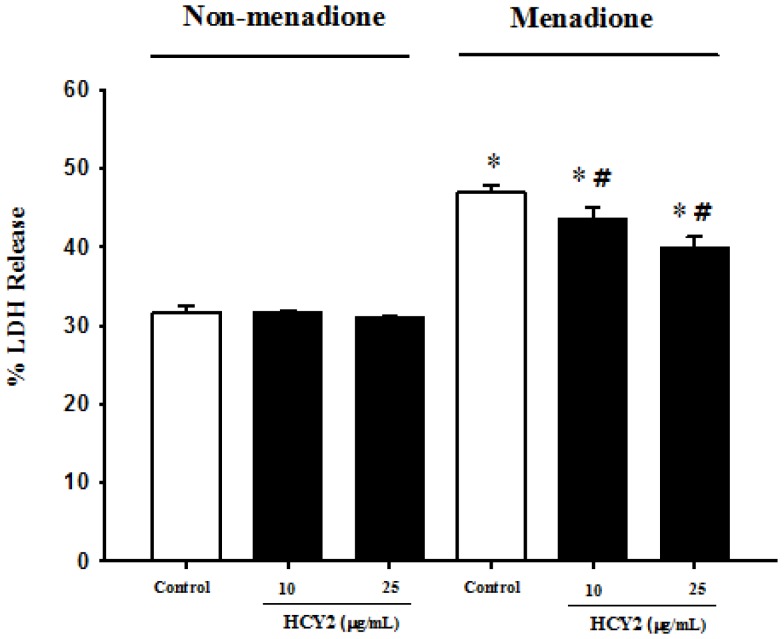
Effects of HCY2 on menadione-induced cytotoxicity in H9c2 cells. Cells were pre-incubated with HCY2 (10 or 25 µg/mL) for 4 h, followed by another 4 h-incubation with or without menadione (12.5 µM). The extent of LDH release was estimated as described in the Experimental Section. Values given are means ± SD, with *n* = 4. * Significantly different from the non-menadione challenged control group (*p* < 0.05); # significantly different from the menadione challenged control group (*p* < 0.05).

**Figure 6:**
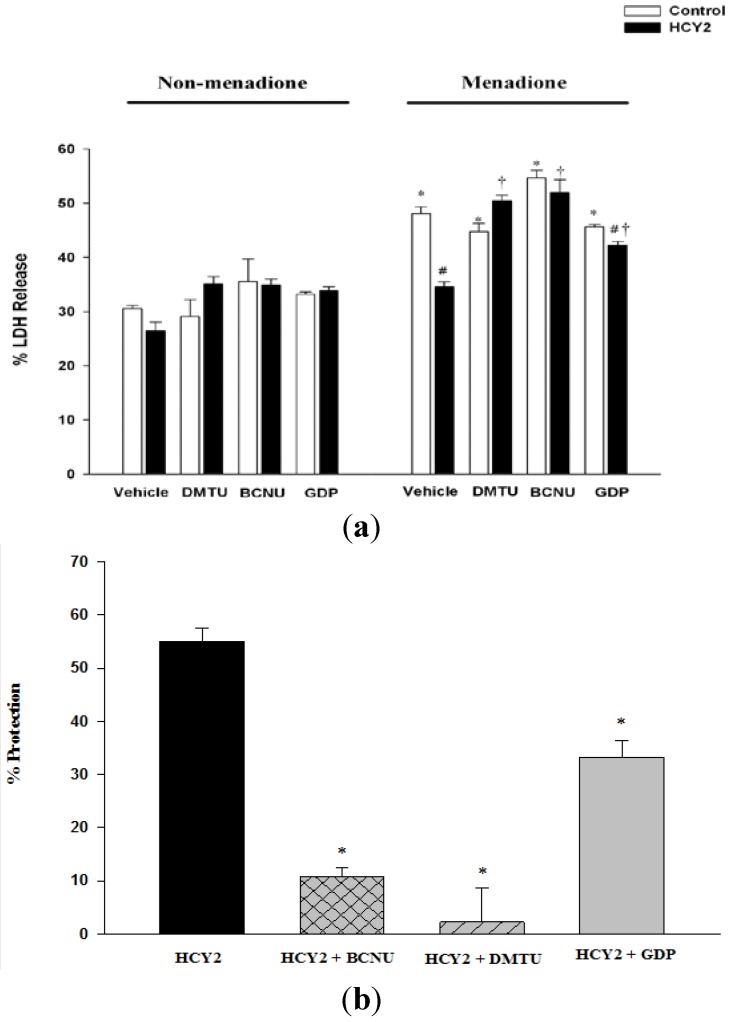
Effects of DMTU, BCNU and GDP on HCY2-induced protection against menadione toxicity in H9c2 cells. Cells were pre-incubated with HCY2 (25 µg/mL) in the absence or presence of DMTU (20 mM), BCNU (50 µM) or GDP (500 µM) for 4 h, followed by another 4 h incubation with menadione (12.5 µM). (**a**) The percentage of lactate dehydrogenase (LDH) release was measured as described in the Experimental Section; and (**b**) The degree of protection against menadione-induced cytotoxicity was estimated as described in the Experimental Section.

To determine the biological significance of the HCY2-induced cellular responses, the effect of HCY2 on menadione-induced oxidant injury in H9c2 cells was examined. As shown in Figure 5, HCY2 at both 10 and 25 µg/mL did not produce any detectable changes in LDH leakage in H9c2 cells. Menadione caused cellular injury in H9c2 cells, as evidenced by the significant increase in the extent of LDH leakage (148%). H9c2 cells were pre-incubated with HCY2 (10 or 25 µg/mL) and subjected to menadione challenge. The result showed that prior exposure to HCY2 significantly suppressed the menadione-induced LDH leakage in a concentration-dependent manner, with the extent of protection at the two tested concentrations of HCY2 being 22% and 42%, respectively. As shown in Figure 6a, DMTU and GDP did not alter the LDH leakage under non-menadione and menadione challenge conditions. Although BCNU did not alter LDH release under non-menadione challenge conditions, it increased the sensitivity of H9c2-treated cells to menadione toxicity. When co-incubated with DMTU or BCNU, HCY2 did not suppress the menadione-induced LDH release as compared with the respective control. In other words, the cytoprotection afforded by HCY2 was completely abrogated by both DMTU and BCNU co-incubation. However, the use of GDP only partially suppressed the cytoprotective effect of HCY2 against menadione-induced cytotoxicity (Figure 6b).

(**a**)* Significantly different from the respective non-menadione control group; # significantly different from the respective manadione control group; † significantly different from the respective HCY2 pre-incubated menadione group (*p* < 0.05); (**b**) * Significantly different from the HCY2 pre-incubated group (*p* < 0.05). Values given are means ± SD, with *n* = 3.

**Figure 7:**
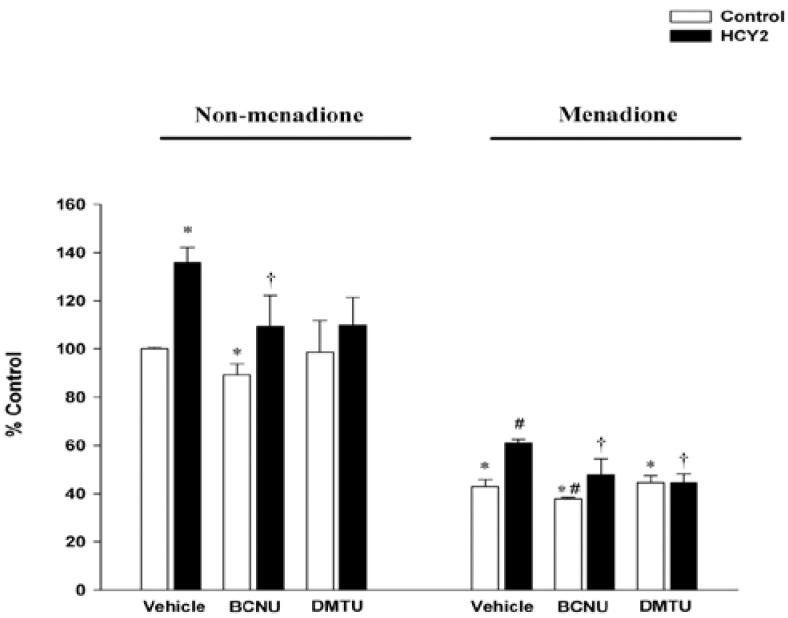
Effects of HCY2 on cellular GSH levels in non-menadione and menadione-challenged H9c2 cells in the absence or presence of DMTU or BCNU. Cells were pre-incubated with HCY2 (25 µg/mL) in the absence or presence of DMTU (20 mM) or BCNU (50 µM) for 4 h, followed by another 4 h-incubation with or without menadione (12.5 µM). Cellular GSH levels were measured under non-menadione or menadione challenged conditions as described in the Experimental Section. Data are expressed as the percentage of non-menadione challenged control value (23.45 ± 0.16 nmol/mg protein). Values given are means ± SD, with *n* = 3. * Significantly different from the non-menadione challenged control group (*p* < 0.05); # significantly different from the menadione challenged control group (*p* < 0.05); † significantly different from the respective HCY2-pre-incubated group (*p* < 0.05)

Figure 7 shows that while menadione-induced cytotoxicity was accompanied by a drastic decrease in cellular GSH levels (57%), HCY2 pre-incubation at 25 µg/mL increased cellular GSH levels in H9c2 cells under both non-menadione and menadione-challenged conditions. While BCNU decreased GSH levels under both non-menadione and menadione-challenged conditions, DMTU did not alter GSH levels when compared with their respective controls. The HCY2-induced increase in GSH levels was completely suppressed by the c o-incubation with BCNU and DMTU when compared with the HCY2 group.

Mitochondria not only serve as the powerhouse of the cell, but they also are the primary source of cellular ROS production [14]. The electron transport chain, in which a number of one-electron transfer reactions occur, has been recognized as the most important site of ROS generation in mitochondria [15]. Although ROS are usually considered as culprits in the pathogenesis of a number of diseases, a compelling body of evidence has demonstrated that low levels of ROS production also play an important role in intracellular redox homeostasis and signal transduction [16].

A previous study in our laboratory has demonstrated the ability of an ethanol extract of Herba Cynomorii to increase mitochondrial ATP-GC, which was associated with the stimulation of pyruvate-supported mitochondrial electron transport in H9c2 cells [10]. In the present study, HCY2, a UA-enriched fraction isolated from an ethanol extract of Herba Cynomorii, was found to stimulate mitochondrial electron transport *in situ* in H9c2 cells. As mitochondrial ROS are unavoidably produced as a consequence of the oxidative phosphorylation process, particularly under conditions of increased electron transport activity [17], the stimulation of mitochondrial electron transport by HCY2 was associated with an increased mitochondrial ROS production. The increase in mitochondrial electron transport was also accompanied by an increase in mitochondrial membrane potential, with the latter inhibiting further electron transport but favoring ROS production [17]. Superoxide anion radicals generated during mitochondrial respiration are capable of activating UCPs on the inner mitochondrial membrane, thereby lowering the membrane potential through dissipation of the proton gradient which is its major determinant [18]. With respect to uncoupled respiration, our preliminary study indicated that HCY2 increased the expression of UCP3 in cultured C2C12 cells (unpublished data). The uncoupling effect of UCPs could therefore be inhibited by purine nucleotide diphosphate, such as GDP [18]. Under the present experimental conditions, GDP, was found to reverse the decrease in mitochondrial membrane potential induced by HCY2, suggesting the involvement of activation of mitochondrial UCPs in the process. The possible involvement of UCPs in HCY2-induced uncoupled respiration is supported by the finding that GDP can reverse the HCY2-induced decrease in RCR, as observed in the present study [19]. Moreover, the UCP-mediated-mitochondrial uncoupling induced by HCY2 was completely prevented by the co-incubation with DMTU, suggesting that the uncoupling effect of HCY2 may also be related to ROS production.

GSH is regarded as the first line of defense in preventing cellular oxidative damage [20]. In the present study, incubation of H9c2 cells with HCY2 was found to induce a time-dependent cyclic variation in cellular GSH levels, indicative of an up-regulation of glutathione redox cycling. BCNU was used to investigate the role of GR in the glutathione response. The finding of GSH depletion following incubation with BCNU supports the involvement of GSH regeneration in the maintenance of cellular GSH status in H9c2 cells. BCNU co-incubation abrogated the HCY2-induced cyclic changes in cellular GSH in H9c2 cells, suggesting the involvement of GSH regeneration from GSSG in the process. Although DMTU did not affect the cellular GSH level in H9c2 cells, the enhancement of glutathione redox cycling by HCY2 was also attenuated by the co-incubation with DMTU, implicating the involvement of mitochondrial ROS production in the HCY2-induced cellular antioxidant response. Conceivably, the prolonged incubation with HCY2 could lead to a continuous production of a small amount of signaling ROS in association with the increase in mitochondrial electron transport. The increased level of ROS would oxidize and hence deplete cellular GSH, with the resultant activation of GR-catalyzed regeneration of GSH from GSSG. Therefore, the activation of glutathione redox cycling is presumably related to the interplay between HCY2-induced ROS generation and the GR-catalyzed regeneration of GSH. Menadione, at high concentrations, is considered to be a cytotoxic substance that can undergo redox cycling through the catalysis of flavoprotein reductase, resulting in oxidative stress-induced tissue injury [21]. Our study demonstrated that incubation with HCY2 significantly protected against menadione-induced cytotoxicity in H9c2 cells. To determine the role of mitochondrial ROS in protecting against menadione cytotoxicity, a thiol-containing antioxidant, DMTU, was used in this study. Although it would be expected that DMTU would protect against cytotoxicity by scavenging free radicals produced from menadione, the result showed that DMTU did not produce any detectable effect on menadione-induced LDH leakage or cellular GSH depletion in H9c2 cells, which was consistent with previous experimental data in our laboratory [22]. Indeed, Kossenjans *et al.* demonstrated that exposure to menadione at much higher concentrations (100–200 µM) for 10 h induced drastic LDH leakage in bovine heart microvascular endothelial cells, which was preventable by DMTU [23]. By contrast, another research group reported that DMTU could not attenuate menadione (15 µM, 4 h)-induced LDH release and cellular GSH depletion in endothelial cells [24], in which the experimental conditions were similar to those in the present study. The differential effects of DMTU on menadione cytotoxicity may be due to the fact that prolonged exposure to high concentrations of menadione produced free radicals in larger amounts than that of a brief exposure to lower concentrations. By virtue of its free radical scavenging activities, DMTU could reduce the extent of cellular injury induced by high concentrations of menadione. However, DMTU did not attenuate cellular injury caused by low concentrations of menadione presumably because it was unable to reduce the extent of GSH depletion which is detrimental to cell survival.

The protection against oxidant injury afforded by HCY2 was completely abrogated by DMTU co-incubation, suggesting the involvement of ROS-mediated processes in the cytoprotective effect. The HCY2-induced increase in cellular GSH levels was also attenuated by DMTU under both control and menadione-intoxicated conditions. To define the role of GSH regeneration in the cytoprotection afforded by HCY2, the GR inhibitor (BCNU) was used as an experimental tool. The enhanced susceptibility of BCNU-pre-incubated cells to menadione cytotoxicity supports the role of GSH regeneration in maintaining cell survival under conditions of oxidative stress. BCNU was also found to decrease GSH levels following menadione exposure when compared with corresponding controls. The protective effect of HCY2 on menadione-induced toxicity was largely abolished by the co-incubation with the GR inhibitor. This is consistent with the observation that the decrease in the extent of protection against menadione toxicity afforded by HCY2 pre-incubation was paralleled by an attenuation in the HCY2-induced GSH antioxidant response by BCNU. The results therefore suggest that HCY2-induced glutathione redox cycling plays a determining role in protecting against menadione cytotoxicity in H9c2 cells. In addition, GDP was used to delineate the role of mitochondrial uncoupling in the cytoprotection afforded by HCY2. Our results indicated that GDP did not affect menadione-induced LDH leakage. The co-incubation with GDP partially abolished the cytoprotection afforded by HCY2 against menadione challenge, implicating the involvement of mitochondrial uncoupling in this process.

Our preliminary study indicated that UA, the major component in HCY2, produced similar effects on ROS production and cytoprotection as the HCY2 preparation (unpublished data). UA has also been found to be a potential cardioprotective agent, which may act at least in part through the uncoupling of mitochondrial oxidative phosphorylation [25]. However, one cannot exclude the possibility that other constituents in HCY2 may also exert antioxidant effects that can protect against menadione cytotoxicity. In this regard, further comparative studies on mechanisms underlying the cytoprotective effect of UA and HCY2 on oxidant injury in H9c2 cells are warranted.

Taken collectively, our results indicate that the cytoprotection afforded by HCY2 against menadione toxicity is at least partly contributed to by UCP-mediated mitochondrial uncoupling, which is an event secondary to increased ROS production. More importantly, the elicitation of a glutathione antioxidant response, which is primarily caused by an increase in ROS production, plays a crucial role in the cytoprotective action of HCY2.

## 3. Experimental

### 3.1. Chemicals

Dulbecco’s Modified Eagle’s Medium (DMEM) and fetal bovine serum (FBS) were purchased from Gibco BRL Life Technologies (Grand Island, NY, USA). 2',7'- dichlorofluorescein diacetate (DCFDA) was purchased from Fluka (Buchs, Switzerland). Other chemicals were purchased from Sigma Chemical (St Louis, MO, USA). All chemicals were of analytical grade.

### 3.2. Herbal Material and Extraction

Herba Cynomorii was purchased from Lee Hoong Kee, Ltd., a local Hong Kong-based herbal dealer. The herb was authenticated by the supplier and a voucher specimen (HKUSTY01001) was deposited in the Division of Life Science, the Hong Kong University of Science and Technology (HKUST). An UA-enriched active fraction, HCY2, was isolated from an ethanol extract of Herba Cynomorii as previously described [9]. The content of UA in HCY2 was determined by using an HPLC-UV method. HPLC analysis was performed using an Agilent RRLC 1200 series system (Agilent, Waldbronn, Germany). HCY2 (dissolved in acetonitrile) was separated on a Waters Atlantis C18 column (5 µm id, 4.6 mm × 150 mm). The mobile phase was composed of acetonitrile (solvent A) and 0.1% formic acid in water (solvent B), using an isocratic gradient 78% (solvent A) for 50 min. The detection wavelength was set at 210 nm. The quantitation of UA in the HCY2 fraction was determined from a calibration curve. A standard stock solution of UA (5 mg/mL) was prepared in methanol. Serial dilutions with methanol were made of each stock solution to prepare standard solutions of 50, 100, 250, 500 and 1000 μg/mL; 10 µL of each standard solution was used for analysis and the construction of a standard calibration curve. The calibration curve of UA was linear and the regression equation of the peak area (y) as a function of concentration (x) was: y = 473.2x + 532.6 (*r* = 0.999). The concentration of UA in HCY2 was estimated to be 74.8% (w/w). (HPLC-UV chromatography was provided as supplementary document)

### 3.3. Cell Culture

H9c2 cells, a permanent cell line derived from embryonic BD1X rat heart tissue, were purchased from American Type Culture Collection. The cells were cultured as monolayers in DMEM supplemented with 10% (v/v) FBS. The medium contained 4.5 g/L of glucose (4.5 g/L) and glutamine (4.5 mM), supplemented with NaHCO_3_ (17 mM), penicillin (100 IU/mL) and streptomycin (100 μg/mL). All cells were grown under an atmosphere of 5% CO_2_ in air (v/v) at 37 °C. The medium was replaced every 2–3 days. A stock of cells was grown in a 75 cm^2 ^culture flask and split before confluence at a subcultivation ratio of 1:10.

### 3.4. Measurement of Mitochondrial Electron Transport in H9c2 Cells

H9c2 cells were seeded in 75 cm^2^ culture flasks and allowed to grow to 70%–80% confluence prior to pre-incubation with the herbal extract. HCY2 (dissolved in DMSO) was added to the medium to achieve the desired final concentrations (DMSO < 0.2%, v/v). After a 4-h incubation with HCY2 (10 or 25 µg/mL), the HCY2-containing medium was aspirated and cells were trypsinized and permeabilized with digitonin (50 μg/mL). A mitochondria-rich fraction was isolated as described [26]. The measurement of electron transport in isolated mitochondria, which is based on the reduction of MTT, was performed using modified methods as described in [27]. An aliquot (40 µL) of the mitochondria-rich fraction was mixed with 100 µL each of 15 mM pyruvate, 7.5 mM malate and 0.42 mg/mL MTT. The reaction mixture was incubated at 37 °C for 10 min with gentle shaking. After the incubation, the reaction was terminated by the addition of 100 µL lysis buffer (10%, w/v, sodium dodecyl sulfate and 45% dimethylformamide, adjusted to pH 4.7 with glacial acetic acid). After standing for 5 min, the absorbance at 570 nm of the reaction mixture was measured with a multi-titer plate reader (Bio-Rad, Hercules, CA, USA). Data were normalized to a mean control value of non-herbal extract pre-incubated samples and expressed as a percentage of control.

### 3.5. Measurement of Mitochondrial ROS Production in H9c2 Cells

H9c2 cells were seeded in 75 cm^2^ culture flasks and allowed to grow to 70%–80% confluence prior to pre-incubation with the herbal extract. After a 4-h incubation with HCY2 (10 or 25 µg/mL), a mitochondria-rich fraction was isolated as described above. An aliquot (50 μL) of mitochondria-rich fraction (adjusted to 1 mg protein/mL) was added to a well of a black multi-titer plate and incubated with 60 μL of DCFDA (17.5 μM in incubation buffer) for 10 min at 37 °C. After the incubation, an aliquot (50 μL) of substrate mixture (20 mM pyruvate and 10 mM malate) was added to each well. Fluorescence emission (excitation 485 nm and emission 535 nm) was monitored every 5 min to until 30 min at 37 °C. ROS generation was calculated from fluorescence intensities after subtracting the fluorescence value of a blank sample containing only incubation buffer, substrate solution and DCFDA. The extent of ROS generation over the 30-min period was estimated by computing the area under the curve (AUC) of a graph plotting fluorescence intensity against time (0 to 30 min). To investigate the effect of an antioxidant on HCY2-induced ROS production, cells were co-incubated with dimethylthiourea (DMTU 20 mM) and HCY2 (25 µg/mL) for 4 h and subjected to the measurement of mitochondrial ROS production as described above. The AUC of herbal extract pre-incubated samples were expressed as a percentage of the non-herbal extract pre-incubated controls.

### 3.6. Measurement of Mitochondrial Membrane Potential in H9c2 Cells

H9c2 cells were seeded (1.0 × 10^4^ cells) on a 96-well black multi-titer plate with a clear bottom and were grown for 48 h before use. Cells were washed with phosphate buffered saline-A (PBS-A) twice and loaded with 100 µL JC-1 (Sigma Chemical., St Louis, MO, USA) dye solution (20 µM in PBS-A) for 10 min under an atmosphere of 5% CO_2_ in air (v/v) at 37 °C. After staining with JC-1, the cells were washed with PBS-A twice and incubated with HCY2 (10 or 25 µg/mL) in the presence or absence of GDP (500 µM). Carbonyl cyanide-*p*-trifluoromethoxyphenylhydrazone (FCCP, 100 µM), a chemical uncoupler, was used as a positive control in the measurement. The accumulation of JC-1 dye in mitochondria was quantified by red fluorescence intensity (excitation 527 nm and emission 590 nm), which was monitored every 2 min to up to 40 min at 37 °C. The changes in mitochondrial membrane potential were expressed as the percentage of initial level of the corresponding control. 

### 3.7. Measurement of Mitochondrial Respiration in H9c2 Cells

Mitochondrial respiratory activity was measured polarographically with a Clark-type oxygen electrode (Hansatech Instruments, Norfolk, UK) as described [9]. H9c2 cells were seeded (2.0 × 10^6^ cells) in 100 mm culture plate and pre-incubated with HCY2 (25 µg/mL) for 4 h at 37 °C. After the incubation, cells were collected by trypsinization and suspended in assay buffer (120 mM KCl, 5 mM KH_2_PO_4_, 2 mM EGTA, 10 mM HEPES, 0.1 MgCl_2_, 0.5% BSA, pH 7.4) and kept at 37 °C. An aliquot (1 mL) of suspended cells (1.5 × 10^6^ cells/mL) was placed in an air-tight liquid-phase oxygen electrode chamber. The system was maintained at 30 °C using a constant temperature water-jacketing system. Following equilibration, a non-ionic detergent, digitonin (50 μg/mL), was added and incubated for 3 min to permeabilize cell membranes. This was followed by the addition of pyruvate (5 μM), malate (2.5 μM) and ADP (60 μM) to intiate mitochondrial state 3 respiration. Mitochondrial state 4 respiration was then induced by the addition of the specific complex V inhibitor, oligomycin (1 mg/mL). To investigate the role of UCPs in mitochondrial respiration, guanosine diphosphate (GDP, 500 µM) was added following the attainment of a steady state 4 respiration. For HCY2 pre-incubated cells, the rate of mitochondrial respiration was normalized to a mean control value from non-herbal extract pre-incubated cells and expressed as a percent of control.

### 3.8. Measurement of Cellular GSH Levels in H9c2 Cells

H9c2 cells were seeded (3.75 × 10^4^ cells/well) in 12-well culture plates. After stable attachment, cells were pre-incubated with HCY2 (25 µg/mL) in the absence or presence of DMTU (20 mM) or bis-chloroethylnitrosourea (BCNU, 50 µM, a specific inhibitor of GR) at indicated time intervals at 37 °C. Following the commencement of incubation, GSH levels were determined at increasing time intervals (4–24 h). Cellular GSH levels were determined enzymatically using DTNB [5,5'-dithiobis-(2-nitrobenzoic acid)] and GR using a method modified from Griffith (1980) [28]. 

### 3.9. Menadione-Induced Cytotoxicity in H9c2 Cells

H9c2 cells used in the experiment were seeded at 3.75 × 10^4^ cells in 12-well culture plate. After stable attachment, cells were pre-incubated with HCY2 for 4 h at 37 °C. After the incubation with herbal extract, the herbal extract-containing medium was aspirated and the cells were incubated with menadione-containing medium (12.5 μM) for another 4 h at 37 °C. For the non-menadione-challenged (non-Men) groups, the same volume of vehicle (*i.e.*, ethanol) was added to the culture medium. After the menadione challenge, lactate dehydrogenase (LDH) activities in cell lysates and culture medium were measured in unchallenged and challenged cells, with or without herbal extract pre-incubation. The culture medium was collected and stored in a 1.5 mL micro-centrifuge tube at 4 °C. Cells were then washed with PBS-A, and an aliquot (300 μL) of lysis buffer [0.1% (w/v) Triton X-100 in PBS] was added, and the mixture was incubated at 4 °C for 10 min prior to the LDH assay. LDH activity was measured as described by Li *et al.* [29]. The extent of LDH release was estimated as follows: [(LDH released into the medium in unchallenged or challenged cells) / (cellular LDH + LDH in the medium of unchallenged or challenged cells) × 100%. The degree of protection against menadione-induced cytotoxicity was estimated using the equation: [(LDH_Men Control_ − LDH_non Men Herbal_) − (LDH_Men Herbal_ − LDH_non Men Herbal_) / (LDH_Men Control_ − LDH_non Men Herbal_) × 100%].

### 3.10. Statistical Analysis

All data were expressed as mean ± standard deviation (SD). Data were analyzed by one-way analysis of variance (one-way ANOVA) and intergroup differences were detected by the Scheffe method (single-step multiple comparison), with a value of *p* < 0.05.

## 4. Conclusions

We can conclude that an ursolic acid-enriched active fraction derived from Herba Cynomorii protects against menadione cytotoxicity in H9c2 cells. The cytoprotective effect may, at least in part, be attributed to an increase in mitochondrial uncoupling and glutathione redox cycling through the intermediacy of mitochondrial ROS production.

## Supplementary Materials

Supplementary materials can be accessed at: http://www.mdpi.com/1420-3049/19/2/1576/s1.
