# Supplementary figures and images for: Ursolic Acid-Enriched Herba Cynomorii Extract Induces Mitochondrial Uncoupling and Glutathione Redox Cycling Through Mitochondrial Reactive Oxygen Species Generation: Protection Against Menadione Cytotoxicity in H9c2 Cells
Source: Molecules. 2014 Jan 27;19(2):1576–91. doi: 10.3390/molecules19021576 (PMC6271489; doi:10.3390/molecules19021576)

## Supplementary Materials

**Figure S1.** HPLC-UV chromatogram of ursolic acid and HCY2.

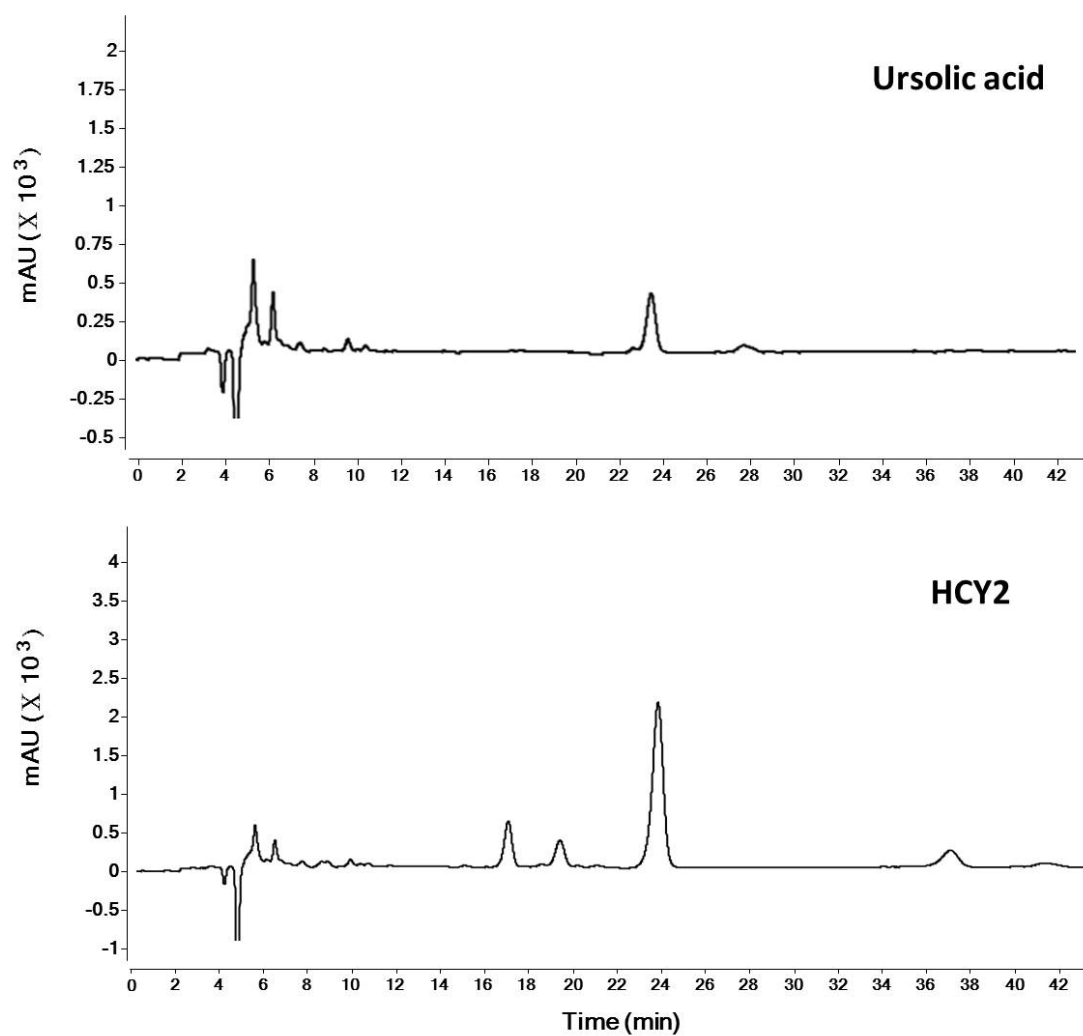

Supplement: Supplementary file 1 [file molecules-19-01576-s001.pdf]
